# Adiponectin Deficiency Suppresses Rhabdomyosarcoma Associated with Gut Microbiota Regulation

**DOI:** 10.1155/2021/8010694

**Published:** 2021-01-23

**Authors:** Jiao Peng, Jia-yue Wang, Hai-feng Huang, Ting-ting Zheng, Jie Li, Li-jun Wang, Xiao-chi Ma, Hai-tao Xiao

**Affiliations:** ^1^Department of Pharmacy, Peking University Shenzhen Hospital, Shenzhen, China; ^2^School of Pharmaceutical Sciences, Health Science Center, Shenzhen University, Shenzhen, China; ^3^The High Efficacy Application of Natural Medicinal Resources Engineering Center of Guizhou Province, School of Pharmaceutical Sciences, Guizhou Medical University, Guiyang, China; ^4^School of Pharmaceutical Science, Chongqing Medical University, Chongqing, China; ^5^Shenzhen Key Laboratory for Drug Addiction and Medication Safety, Department of Ultrasound, Peking University Shenzhen Hospital, Shenzhen PKU-HKUST Medical Center, Shenzhen, China; ^6^Department of Laboratory Medicine, Peking University Shenzhen Hospital, Shenzhen, China

## Abstract

The gut microbiota is very important in the initiation, progression, and dissemination of cancer, and the regulation of microbiota has been employed as a novel strategy to enhance the effect of immunotherapy. Adiponectin (APN), an adipocyte-derived hormone, plays a vital role in regulating the immune response of innate immune cells. The deficiency of APN inhibits rhabdomyosarcoma growth. However, whether this function is associated with regulating gut microbiota remains unknown. To investigate, we performed 16S ribosomal RNA (rRNA) gene sequencing on the fecal microbiome of APN gene knockout mice to determine whether APN deletion affects the gut microbiota. We found APN deficiency alters gut microbial functions involved in metabolism, genetic information processing, and cellular processes. In addition, a decreased abundance of *Bacteroides* and an increased abundance of *Prevotella* and *Helicobacter* were observed in rhabdomyosarcoma-bearing APN knockout mice; these bacteria were associated with the inhibition of rhabdomyosarcoma growth. These findings suggest that gut microbiota may be a potential target of APN deficiency against rhabdomyosarcoma.

## 1. Introduction

Rhabdomyosarcoma (RMS) is the most common pediatric soft tissue sarcoma, listing among the top 20 most diagnosed cancers in the world [1, 2]. RMS is a typical embryonal tumor in childhood, characterized by skeletal muscle differentiation (WHO STS 2013) [3]. Overall, the incidence of RMS is 4.3 million/year worldwide; two-thirds of all cases are diagnosed before seven years old. Although it occurs mainly in children's age group (0–10 years old), it also has a high incidence in puberty and youth (15–30 years old), but it is extremely rare in adults [3]. Histologically, RMS can be classified into two major histotypes: embryonal rhabdomyosarcoma (ERMS) and alveolar rhabdomyosarcoma (ARMS) [3]. ERMS accounts for about 80% of all cases and usually affects children aged 0–4 years, occurring in the neck, head, and reproductive urinary tract; ARMS, a highly malignant tumor that mainly occurs in teenagers, accounts for about 20% of RMS cases [3]. Currently, the available clinical treatment, including laser surgery, radiation therapy, chemotherapy, immunotherapy, organ sparing, and transoral robotic surgery, is considered the standard treatment of care for RMS. With advancements in the treatment of RMS, the five-year survival rate has increased from 25% to approximately 70% [1, 2]. However, some current treatments for RMS are associated with high costs, increased toxicity, and numerous side effects [1]. There is consequently a clear demand to further explore the pathological mechanism of RMS and develop new therapeutic strategies for RMS.

Adiponectin (APN), a 30-kDa adipokine primarily derived from adipose tissue, plays a vital role in regulating the immune response of innate immune cells [4]. Our previous work found that APN was expressed at the highest level in RMS among four human pediatric sarcomas, namely RMS, hepatoblastoma, nephroblastoma, and neuroblastoma. And in both histotypes of RMS, ARMS has a much higher level of APN expression than ERMS [5]. In addition, we also found that APN deficiency could induce the polarization of tumor-associated macrophages to an M1-like phenotype and inhibit the growth of rhabdomyosarcoma in mice, indicating that APN may be a potential immunotherapy target for RMS [5]. Recent studies have claimed that gut microbiota is a key player for the development of tumors and regulation of gut microbiota emerged as a novel strategy to improve the efficacy of immunotherapy [6, 7]. More recently, Blanca Grases-Pintó and his colleagues reported that supplement with APN resulted in the decrease of *Roseburia* genus and the increase of *Enterococcus* genus in the intestine of suckling rats, suggesting a modulatory role of APN in gut microbiota [8]. Therefore, in this study, we hypothesized that APN deficiency against rhabdomyosarcoma is closely associated with altering the gut microbiota. Here, we first investigated the effects of APN deficiency on gut microbiota in rhabdomyosarcoma-bearing mice and found APN deficiency against rhabdomyosarcoma was positively related to the relative abundance of *Prevotella* and *Helicobacter* and negatively correlated to the relative abundance of *Bacteroides*.

## 2. Material and Methods

### 2.1. Animal Experiments and Sample Collection

The APN^−/−^ mice on the C57BL/6 background were gifted from Prof. AM Xu (The University of Hong Kong). C57BL/6 wild-type mice were obtained from Beijing Vital River Laboratory Animal Technology Co., Ltd. (Beijing, China). All mice were raised in an environment with a temperature of 23 ± 2°C and a 12 h light/dark cycle. The offspring of C57BL/6 wild-type mice and APN^−/−^ mice were marked with ear tags and raised together in the same cage. All experimental protocols were approved by the Animal Ethics Committees of Shenzhen University in accordance with the “Institutional Guidelines and Animal Ordinance” (Health Science Center, Shenzhen University, Registration No. 2018020).

Inbred 6–8-week-old male offspring of C57BL/6 wild-type mice and APN^−/−^ mice were selected and used in the experiments reported herein. The mouse soft tissue sarcoma was established as described in our previous study [5]. In brief, 1 × 10^5^ MN/MCA1 cells were inoculated into the caudal thigh muscle of mice. The tumor mass was monitored, and the size was recorded twice a week. On day 21, the mice were sacrificed and fresh cecum faeces were collected.

### 2.2. Histologic Sections

Rhabdomyosarcoma tumor tissues were fixed in formalin and embedded in paraffin. Five micrometers of sections were made and stained with hematoxylin-eosin (H&E).

### 2.3. Fecal 16S rRNA Analysis

Fresh cecum faeces were collected and weighed. The total DNA of faeces was extracted using a DNA extraction kit (TIANGEN, China). The quality and quantity of DNA were measured by the ratios of 260 nm/280 nm and 260 nm/230 nm, respectively. Subsequently, each extracted DNA was used as a template, and their V3–V4 region of 16S rRNA genes of distinct regions were amplified with specific primers (515F: 5′-GTGCCAGCMGCCGCGGTAA-3′, 806R: 5′-GGACTACHVGGGTWTCTAAT-3′). All Polymerase Chain Reaction (PCR) reactions were performed using Phusion®High-Fidelity PCR Master Mix (New England Biolabs). PCR products were mixed with 1× loading the same buffer at the same volume (including SYBR green) and detected by 2% agarose gel electrophoresis. Further experiments were carried out using samples with sizes between 280 and 320 bp. The TruSeq®DNA PCR-Free Sample Preparation Kit (Illumina, United States) was used to generate sequencing libraries. The library quality was evaluated on the Qubit@ 2.0 Fluorometer (Thermo Scientific) and Agilent Bioanalyzer 2100 system. Finally, the library was sequenced on the Ion S5TM XL platform (Thermo Fisher Scientific, United States). For the bioinformatics analysis, the data processing was followed as previously reported protocol [9, 10]. Briefly, the raw fast files were filtered using Cutadapt (V1.9.1). The OTUs were clustered with a cutoff value of 97% similarity using UPARSE (version 7.0.1001), and chimeric sequences were removed using UCHIME. The community composition analysis was performed and classified using the RDP classifier (http://rdp.cme.msu.edu/) based on SILVA ribosomal RNA gene database.

### 2.4. Statistical Analysis

The results were statistically analyzed using the GraphPad Prism 5 software (San Diego, CA). The data were presented as the mean ± standard error of the mean (SEM). The two-tailed Student's *t*-test was conducted to analyze the differences between the two groups. The statistical analyses were considered significant at *p* < 0.05. Also, the Wilcoxon signed-rank test was applied for linear discriminant analysis of taxa LDA scores and PICRUSt analysis of biological metabolism pathways.

## 3. Results

### 3.1. APN Deficiency Suppresses the Growth of Rhabdomyosarcoma in Mice

**Figure 1:**
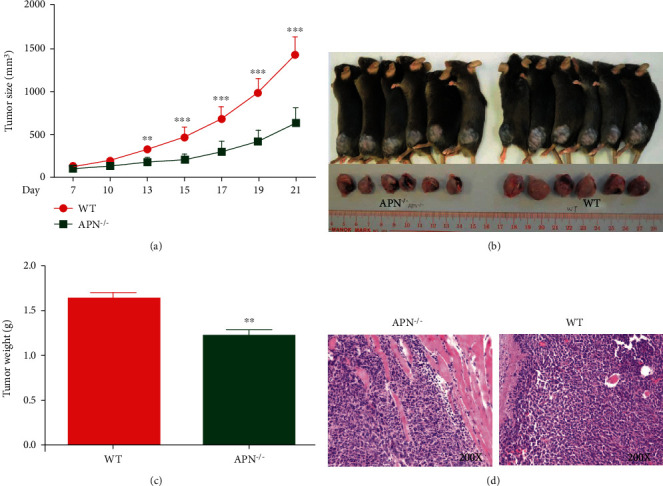
The effects of APN deficiency on rhabdomyosarcoma growth in mice: (a) tumor growth curve; (b) photograph of tumors dissected from APN^−/−^ and wild-type mice; (c) mass of tumors dissected from APN^−/−^ and wild-type mice; (d) H&E staining of tumors dissected from APN^−/−^ and wild-type mice. Data are expressed as the mean ± SEM (*n* = 6). ^∗∗^*p* < .01, compared with the wild-type group.

To evaluate the effect of APN in the development of rhabdomyosarcoma, MN/MCA1 cells were injected into both APN^−/−^ and wild-type mice. During administration, the tumor size was measured every other day. Compared with the wild-type group, the tumor growth in the APN^−/−^ group was significantly suppressed and tumor volumes of the APN^−/−^ group were much smaller on day 13 and after (Figure 1(a)). On day 21, the rhabdomyosarcoma-bearing mice were sacrificed, and tumors were collected to be photographed and weighted. The tumor size of the APN^−/−^ group was smaller than that of the wild-type group (Figure 1(b)), and the tumor mass of the APN^−/−^ group was also much lesser than that of the wild-type group (Figure 1(c)). After dissection, histological staining with H&E was performed. As shown in Figure 1(d), compared to the tumor dissected from APN^−/−^ mice, extensive tumor invasion and muscle degradation with lower immune cell infiltration were observed in the tumor section of the wild-type mice. These data indicate that APN deficiency suppresses rhabdomyosarcoma growth.

### 3.2. APN Deficiency Alters the Diversity of Gut Microbiota in Rhabdomyosarcoma-Bearing Mice

**Figure 2:**
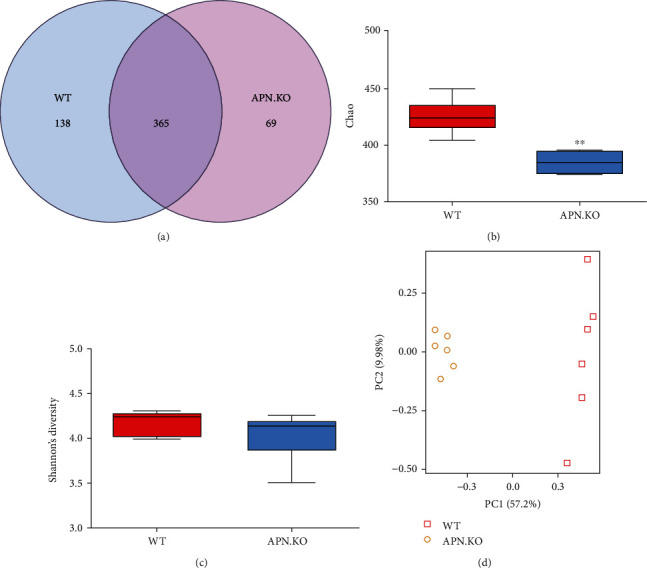
The effects of APN deficiency on the diversity of gut microbiota in rhabdomyosarcoma-bearing mice: (a) Venn diagram for OTUs; (b) Chao diversity; (c) Shannon's richness index; (d) score plot for principal component analysis of OTUs. Data are expressed as the mean ± SEM (*n* = 6). ^∗∗^*p* < .01, compared with the wild-type group.

Since the previous study showed that APN might exhibit a modulatory role in gut microbiota [8], we examined the effect of APN deficiency on the changes of gut microbial communities of rhabdomyosarcoma-bearing mice. In 16S rRNA sequencing analysis, 572 operational taxonomic units (OTUs) were picked in fecal bacteria of all rhabdomyosarcoma-bearing mice in this study. The Venn diagrams showed that APN^−/−^ mice had a 15.89% difference in OTUs compared to wild-type mice (Figure 2(a)). To assess the structure of the gut microbial community, their richness and evenness were calculated (Figures 2(b) and 2(c)). The diversity measured using the Chao diversity index showed a significantly decreased value in the group of APN^−/−^ mice compared with the group of wild-type mice, whereas the diversity measured by Shannon's richness index showed no difference between the two groups. These data suggest that APN deficiency mainly affected the abundance of colonic bacteria rather than their species in the gut of rhabdomyosarcoma-bearing mice. Additionally, the PCA score plot showed a clear separation between rhabdomyosarcoma-bearing wild-type and APN knockout mice, suggesting that APN deficiency significantly affects the structure and composition of gut microbiota in rhabdomyosarcoma-bearing mice (Figure 2(d)).

### 3.3. APN Deficiency Alters the Abundance of Gut Microbiota in Rhabdomyosarcoma-Bearing Mice

**Figure 3:**
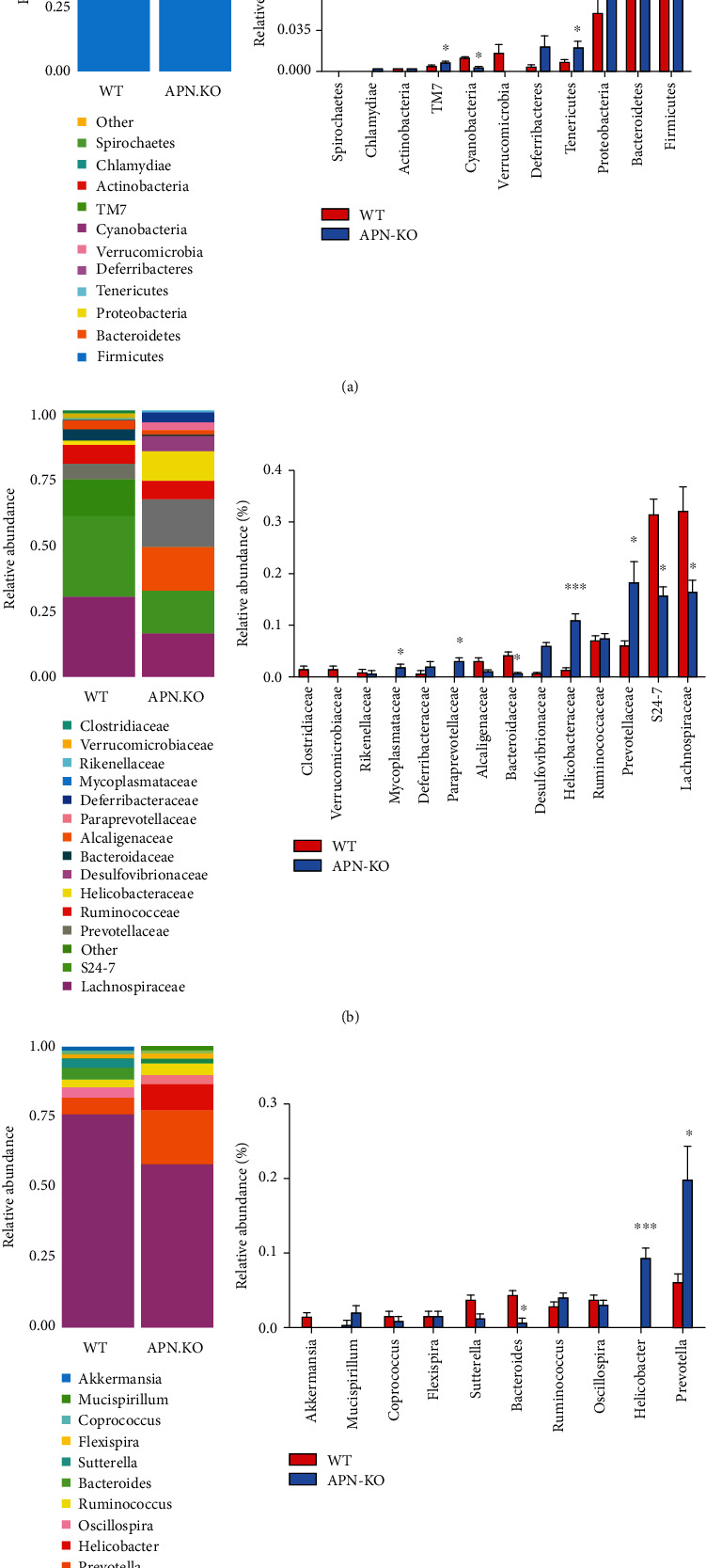
The effects of APN deficiency on the abundance of gut microbiota in rhabdomyosarcoma-bearing mice. (a) The abundance of gut microbiota at the phylum level. (b) The abundance of gut microbiota at the family level. (c) The abundance of gut microbiota at the genus level. Data are expressed as the mean ± SEM (*n* = 6). ^∗^*p* < .05, ^∗∗^*p* < .05, ^∗∗∗^*p* < .001, compared with the wild-type group.

To assess specific changes in the fecal microbiota, the relative abundances of the predominant taxa identified in the two groups were compared (Figure 3). The composition of gut microbiota was significantly different at all taxonomic levels. At the phylum level, the community from rhabdomyosarcoma-bearing APN knockout mice showed a remarkable increase in the relative abundance of *Proteobacteria* (*p* < .001), *Tenericutes* (*p* < .05), *Deferribacteres*, and *TM7* (*p* < .05) and a dramatic decrease in the relative abundance of *Cyanobacteria* (*p* < 0.05) and *Verrucomicrobia* in comparison with those of rhabdomyosarcoma-bearing wild-type mice (Figure 3(a)). At the family level, the fecal microbiota community was occupied by *S24-7*, *Lachnospiraceae*, and *Prevotellaceae* in all groups. Compared to rhabdomyosarcoma-bearing wild-type mice, the fecal microbiota community from rhabdomyosarcoma-bearing APN knockout mice showed a dramatic decrease in the relative abundance of *S24-7* (*p* < .05), *Lachnospiraceae* (*p* < .05), and *Bacteroidaceae* (*p* < .05) and a significant increase in the relative abundance of *Prevotellaceae* (*p* < .05), *Helicobacteraceae* (*p* < .001), *Paraprevotellaceae* (*p* < .05), and *Mycoplasmataceae* (*p* < .05) (Figure 3(b)). At the genus level, the fecal microbiota was occupied by *Prevotella* in all groups. The fecal microbiota community from rhabdomyosarcoma-bearing APN knockout mice showed a remarkable increase in the relative abundance of *Prevotella* (*p* < .05) and *Helicobacter* (*p* < .001) and a significant decrease in the relative abundance of *Bacteroides* (*p* < .05) (Figure 3(c)). Taken together, these results show that APN deficiency alters the abundance of gut microbiota in rhabdomyosarcoma-bearing mice.

### 3.4. APN Deficiency Alters the Phylotypes of Gut Microbiota in Rhabdomyosarcom-Bearing Mice

**Figure 4:**
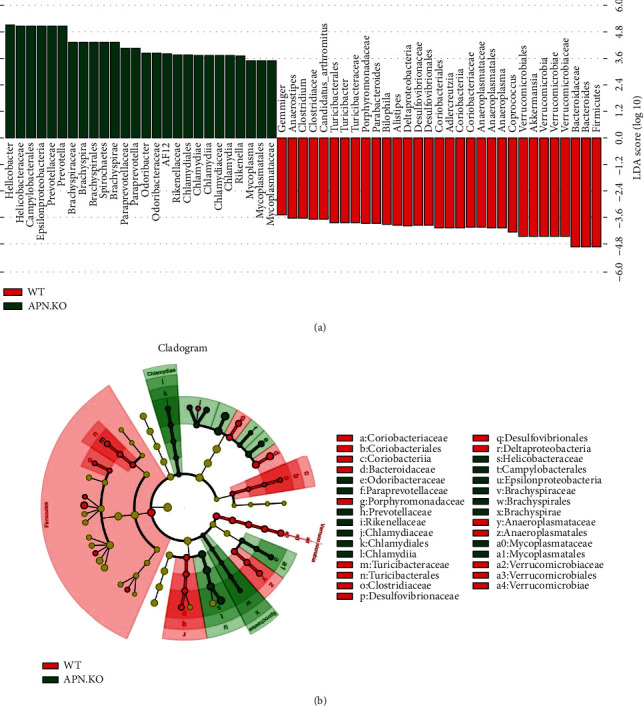
The effects of APN deficiency on the phylotypes of gut microbiota in rhabdomyosarcoma-bearing mice. (a) Linear discriminant analysis (LDA) effect size indicated differences in phyla and genera between the groups WT and APN^−/−^ groups (taxa with LDA score > 2 and significance of *p* < .05 determined by Wilcoxon signed-rank test (*n* = 6)). (b) A cladogram representation of taxa enriched in APN^−/−^ mice (green) microbiota and taxa enriched in WT mice (red) microbiota.

To identify the significantly altered bacteria in each group, LEfSe analysis was performed. The histogram reflected the linear discriminant analysis (LDA) scores calculated by the features at the OTU level, as shown in Figure 4(a). The relative abundance of taxonomic groups with an LDA score greater than 10^4.8^ was summed for the rhabdomyosarcoma-bearing APN knockout mice (*Helicobacter*, *Helicobacteraceae*, *Campylobacterales*, *Epsilonproteobacteria*, *Prevotellaceae*, and *Prevotella*) and rhabdomyosarcoma-bearing wild-type mice (*Firmicutes*, *Bacteroides*, and *Bacteroidaceae*).  Figure 4(b) depicts the evolution of this taxa with LDA values > 2.0. It showed the most differentially abundant taxa enriched in microbiota with green for rhabdomyosarcoma-bearing APN knockout mice and red for rhabdomyosarcoma-bearing wild-type mice. The diameter of each circle is proportional to its richness. The APN deficiency showed a significant decrease in the phylum of *Coriobacteriaceae*, *Bacteroidaceae*, *Porphyromonadaceae*, *Turicibacteraceae*, *Clostridiaceae*, *Desulfovibrionaceae*, *Anaeroplasmataceae*, and their related subcategories and a greater abundance of the phylum of *Mycoplasmataceae*, *Odoribacteraceae*, *Paraprevotellaceae*, *Prevotellaceae*, *Rikenellaceae*, *Chlamydiaceae*, *Helicobacteraceae*, *Brachyspiraceae*, and their related subcategories when compared with the wild-type group.

### 3.5. APN Deficiency Alters Gut Microbial Function in Rhabdomyosarcoma-Bearing Mice

**Figure 5:**
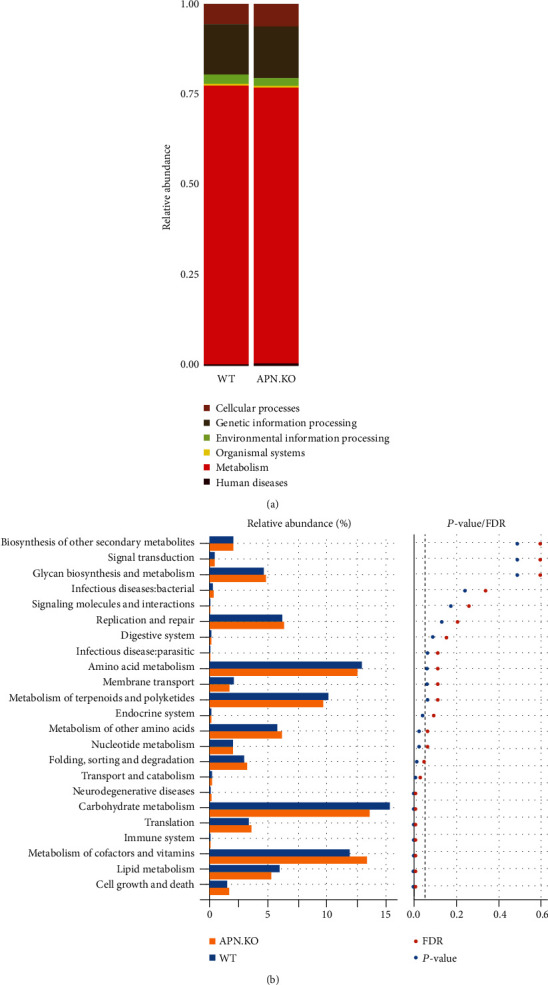
The effects of APN deficiency on gut microbial function in rhabdomyosarcoma-bearing mice. (a) Biological metabolism pathways at level 1. (b) Biological metabolism pathways at level 2 (LDA score > 2 and significance of *p* < .05 determined by Wilcoxon signed-rank test (*n* = 6)).

To examine changes in the gut microbial function of rhabdomyosarcoma-bearing APN knockout mice, PICRUSt was used. As shown in Figure 5(a), six biological metabolism pathways at level 1, including metabolism, genetic information processing, environmental information processing, cellular processes, organismal systems, and human diseases, were extracted from the KEGG database. Among those biological metabolism pathways, metabolism, genetic information processing, and cellular processes dominated, accounting for 75.69%–78.49%, 13.61%–14.86%, and 4.28%–6.49%, respectively. Meanwhile, secondary function analysis of the predictive gene showed that the gene was composed of 23 subfunctions, including membrane transport, carbohydrate metabolism, amino acid metabolism, replication and repair, energy metabolism, translation, cellular processes, and signaling. Within the 23 predictive function categories in level 2 of the KEGG pathway hierarchy, ten predictive function categories, including cell growth and death, metabolism of cofactors and vitamins, immune system, translation, neurodegenerative diseases, transport and catabolism, nucleotide metabolism, metabolism of other amino acids, endocrine system and folding, sorting and degradation, were all significantly increased, and two predicted functional categories—lipid metabolism and carbohydrate metabolism—were significantly decreased in the group of rhabdomyosarcoma-bearing APN knockout mice compared to the group of wild-type mice (Figure 5(b)).

### 3.6. Correlation Analysis of the Relative Abundance of Altered Bacteria and Tumor Size in Rhabdomyosarcoma-Bearing Mice

**Figure 6:**
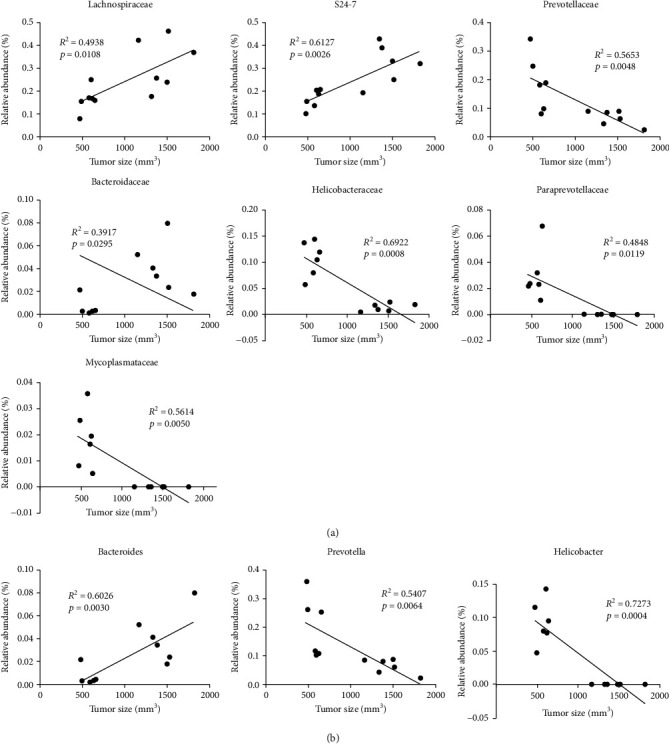
Correlation between the relative abundance of altered bacteria and tumor size in rhabdomyosarcoma-bearing mice: (a) at the family level; (b) at the genus level.

Next, we analyzed the relationship between the abundance of altered bacteria and tumor size in rhabdomyosarcoma-bearing mice. As shown in Figure 6, at the family level, the relative abundance of Lachnospiraceae, S24-7, and Bacteroidaceae were positively correlated to the tumor size, and the relative abundance of Mycoplasmataceae, Paraprevotellaceae, Helicobacteraceae, and Prevotellaceae were negatively correlated to the tumor size (Figure 6(a)). At the genus level, the populations of Bacteroides were positively correlated to the tumor size, while the populations of *Helicobacter* and *Prevotella* were negatively correlated to the tumor size (Figure 6(b)).

## 4. Discussion

The gut microbiota is formed from commensal bacteria and other microorganisms, which can influence physiological functions ranging from the maintenance of local barrier homeostasis to the regulation of metabolism, hematopoiesis, immunity, and other functions systemically [11]. Recently, numerous evidences have revealed that gut microbiota is a key player in the initiation, progression, and dissemination of cancer [12], and it can modulate the response of cancer therapy [6, 7]. In the present study, we found that APN deficiency significantly suppresses the growth of rhabdomyosarcoma, which was related to increasing the relative abundance of *Prevotella* and *Helicobacter*, and correlated to decreasing the relative abundance of *Bacteroides*, suggesting that the inhibitory effect of APN deficiency on rhabdomyosarcoma is associated with the alteration of gut microbiota.

*Prevotella* is a carbohydrate digesting and short-chain fatty acid (SCFA) production bacteria [13]. Recently, SCFAs, including butyrate, are being clinically evaluated as antineoplastic agents because of their suppressive effect on cell growth, cell cycle arrest, differentiation, and/or apoptosis in various tumor cells with favorable safety property to the human. It is well known that SCFAs, such as butyrate, are histone deacetylase (HDAC) inhibitors [14]. Previous studies have documented that HDACs are involved in multiple cellular processes including cell cycle progression, cell differentiation, DNA replication, and genotoxic responses [15]. Moreover, HDACs are found to be increasingly implicated in tumorigenesis [15, 16]. In the present study, the relative abundance of *Prevotella* in rhabdomyosarcoma-bearing APN knockout mice was significantly increased compared to that of rhabdomyosarcoma-bearing wild-type mice, implying an increase of SCFAs from *Prevotella* to inhibit HDACs may partly attribute to the protection of APN deficiency against rhabdomyosarcoma.

*Bacteroides*, members of the human gut microbiota, are closely associated with the efficacy of anticancer immunotherapy. Vetizou et al. reported that the relative abundance of *Bacteroides* in the small intestine mucosa and feces contents was significantly decreased in patients of melanoma treated with CTLA-4 antibodies [17]. Our results also showed that the relative abundance of *Bacteroides* in rhabdomyosarcoma-bearing APN knockout mice was significantly decreased compared with rhabdomyosarcoma-bearing wild-type mice, suggesting APN deficiency suppresses the growth of rhabdomyosarcoma may partly attribute to modulating the abundance of *Bacteroides*. Additionally, *Helicobacter* are Gram-negative bacteria commonly found in the mucosa of the stomach in humans and animals [18]. They can produce many extracellular products, such as urease, HtrA serine proteases (including HtrA1, HtrA2, HtrA3, and HtrA4), and other compounds to enable them to survive in the harsh environment of the stomach [18, 19]. Recently, Rajendran reported that urease is a potent metalloenzyme with inhibitory effects on cancer cell lines through the generation of toxic ammonia to increase the pH of the surrounding medium [20]. In addition, the HtrA proteins are effective modulators to regulate programmed cell death and chemotherapy-induced cytotoxicity [21]. Chien et al. reported that active htrA1 could increase caspase 3/7 activity to induce tumor cell death [22]. In the present study, the relative abundance of *Helicobacter* in rhabdomyosarcoma-bearing APN knockout mice was significantly increased compared with that of the rhabdomyosarcoma-bearing wild-type mice, implying the increase of *Helicobacter* may be beneficial to the protection of APN deficiency, which is associated with increasing the release of extracellular products such as urease and HtrA serine proteases.

The gut microbiota plays a crucial role in host health since it is involved in nutritional, immunologic, and physiological functions. Microbial imbalances caused by gene deficiency in the host might result in altered functions of the gut microbiota. Our results demonstrated significant changes in physiological functions of gut microbiota between APN knockout and wild-type mice. For instance, the genes related to “lipid metabolism” and “carbohydrate metabolism” were found to be decreased while those associated with “nucleotide metabolism” and “metabolism of other amino acids” were found to be increased in the APN knockout group compared to the wild-type group. These findings indicate that the reduced availability of lipids and carbohydrates for cell wall/membrane biogenesis poses a threat to the survival of bacteria. Thus, bacteria might adapt to this situation in other ways, increasing their numbers by increasing the replication rate, thus providing a defense to the resident gut bacteria of the APN knockout mice. On the other hand, reduced availability of lipids and carbohydrates also brought tumor cell death since human cancers have high demands for membrane biogenesis and glycolysis [23, 24], which was consistent with the result that APN deficiency suppresses the growth of rhabdomyosarcoma.

## 5. Conclusion

In summary, our study demonstrated that APN deficiency suppresses the growth of rhabdomyosarcoma, which is closely associated with the alteration of gut microbiota, especially *Prevotella*, *Bacteroides*, and *Helicobacter*. This is the first prospective study using next-generation sequencing to detect the microbial composition of the faeces of rhabdomyosarcoma-bearing APN^−/−^ and wild-type mice, and it highlights that the gut microbiota may be a potential target of APN deficiency against rhabdomyosarcoma.

## Data Availability

The datasets used and/or analyzed during the current study are available from the corresponding author on reasonable request.

## References

[1] Siamayuwa C. E., Nyanga L. K., Chidewe C. (2020). Chemopreventive effects and antioxidant capacity of combined leaf extracts of Sesamum angustifolium (Oliv.) Engl. and Hibiscus articulatus on Rhabdomyosarcoma. *Evidence-based Complementary and Alternative Medicine*.

[2] Chen C., Dorado Garcia H., Scheer M., Henssen A. G. (2019). Current and future treatment strategies for rhabdomyosarcoma. *Frontiers in oncology*.

[3] Gasparini P., Ferrari A., Casanova M. (2019). MiRNAs as players in rhabdomyosarcoma development. *International Journal Of Molecular Sciences*.

[4] Luo Y., Liu M. (2016). Adiponectin: a versatile player of innate immunity. *Journal of Molecular Cell Biology*.

[5] Peng J., Tsang J. Y., Ho D. H. (2015). Modulatory effects of adiponectin on the polarization of tumor-associated macrophages. *International Journal of Cancer*.

[6] Matson V., Fessler J., Bao R. (2018). The commensal microbiome is associated with anti-PD-1 efficacy in metastatic melanoma patients. *Science*.

[7] Guven D. C., Aktas B. Y., Simsek C., Aksoy S. (2020). Gut microbiota and cancer immunotherapy: prognostic and therapeutic implications. *Future Oncology*.

[8] Grases-Pintó B., Abril-Gil M., Castell M. (2019). Influence of leptin and adiponectin supplementation on intraepithelial lymphocyte and microbiota composition in suckling rats. *Frontiers in Immunology*.

[9] He J., He Y., Pan D., Cao J., Sun Y., Zeng X. (2019). Associations of gut microbiota with heat stress-induced changes of growth, fat deposition, intestinal morphology, and antioxidant capacity in ducks. *Frontiers in Microbiology*.

[10] Li Y., Wang S., Sun Y. (2020). Apple polysaccharide protects ICR mice against colitis associated colorectal cancer through the regulation of microbial dysbiosis. *Carbohydrate Polymers*.

[11] Roy S., Trinchieri G. (2017). Microbiota: a key orchestrator of cancer therapy. *Nature Reviews Cancer*.

[12] Carbone C., Piro G., di Noia V. (2019). Lung and gut microbiota as potential hidden driver of immunotherapy efficacy in lung cancer. *Mediators of Inflammation*.

[13] Monk J. M., Lepp D., Wu W., Pauls K. P., Robinson L. E., Power K. A. (2017). Navy and black bean supplementation primes the colonic mucosal microenvironment to improve gut health. *The Journal of Nutritional Biochemistry*.

[14] Tan J., McKenzie C., Potamitis M., Thorburn A. N., Mackay C. R., Macia L. (2014). The role of short-chain fatty acids in health and disease. *Advances in Immunology*.

[15] Chen J. S., Faller D. V., Spanjaard R. A. (2003). Short-chain fatty acid inhibitors of histone deacetylases: promising anticancer therapeutics?. *Current Cancer Drug Targets*.

[16] Stengel K. R., Hiebert S. W. (2015). Class I HDACs affect DNA replication, repair, and chromatin structure: implications for cancer therapy. *Antioxidants & Redox Signaling*.

[17] Vetizou M., Pitt J. M., Daillere R. (2015). Anticancer immunotherapy by CTLA-4 blockade relies on the gut microbiota. *Science*.

[18] Camilo V., Sugiyama T., Touati E. (2017). Pathogenesis of Helicobacter pyloriinfection. *Helicobacter*.

[19] Nightingale T. E., Gruber J. (1994). Helicobacter and human cancer. *Journal of the National Cancer Institute*.

[20] Rajendran R., Pandi A., Ramchary A. (2019). Extracellular urease from Arthrobacter creatinolyticus MTCC 5604: scale up, purification and its cytotoxic effect thereof. *Molecular Biology Reports*.

[21] Chien J., Campioni M., Shridhar V., Baldi A. (2009). HtrA serine proteases as potential therapeutic targets in cancer. *Current Cancer Drug Targets*.

[22] Chien J., Aletti G., Baldi A. (2006). Serine protease HtrA1 modulates chemotherapy-induced cytotoxicity. *The Journal of Clinical Investigation*.

[23] Ganapathy-Kanniappan S., Geschwind J. F. (2013). Tumor glycolysis as a target for cancer therapy: progress and prospects. *Molecular Cancer*.

[24] Cheng C., Geng F., Cheng X., Guo D. (2018). Lipid metabolism reprogramming and its potential targets in cancer. *Cancer Communications*.

